# Depressive disorder vs histrionic personality disorder. Report of a case

**DOI:** 10.1192/j.eurpsy.2021.1986

**Published:** 2021-08-13

**Authors:** F. Cartas Moreno, M. ValverDe Barea

**Affiliations:** 1 Hospital De Úbeda, Unit Mental Health, Ubeda, Spain; 2 Jaén, Complejo Hospitalario Jaén, Jaén, Spain

**Keywords:** PSYCHOPATOLOGY, HISTRIONIC, disorder, Alcohol

## Abstract

**Introduction:**

In daily clinical practice we use to make diagnoses in first consultations, but sometimes it is more complicated, requiring a cross-sectional study of the evolution of the case.In daily clinical practice we use to make diagnoses in first consultations, but sometimes it is more complicated, requiring a cross-sectional study of the evolution of the case.

**Objectives:**

44-year-old woman. Married and mother of one child. She has an hospitalization for alcohol dependence in the context of depressive syndrome. The patient attends the consultation regularly, presenting in the foreground alcohol consumption with evasive characteristics due to hypothymic mood. Many pharmacological approaches are tried with poor tolerance, as well as referral to an alcohol cessation unit. After that, it requires new income where partial disorientation is observed.

**Methods:**

A CT scan is performed and is reported as normal.

**Results:**

In admissions, family-type interventions are performed to reduce accompanying family dysfunction. The evolution is torpid, with the appearance of dysfunctional hysteromorphic personality traits, with childish demands and refusal to go to prescribed consultations. Tendency to confabulation and demonstrative attitudes in the family context, which yield with hospitalization, presenting an absence of disruptive behaviors in the hospital context, but it does seem to present brain alterations due to alcoholism. It is sent home with appropriate indications.

**Conclusions:**

Sometimes, a detailed investigation and follow-up of a case, in this case by way of admission, may result in a different diagnosis than the previous one, which entails a different management.

**Disclosure:**

No significant relationships.

